# Thinking Preferences and Conspiracy Belief: Intuitive Thinking and the Jumping to Conclusions-Bias as a Basis for the Belief in Conspiracy Theories

**DOI:** 10.3389/fpsyt.2020.568942

**Published:** 2020-09-18

**Authors:** Nico Pytlik, Daniel Soll, Stephanie Mehl

**Affiliations:** ^1^Department of Psychiatry and Psychotherapy & Center for Mind, Brain and Behavior (MCMBB), Philipps-University, Marburg, Germany; ^2^Department of Health and Social Work, Frankfurt University of Applied Sciences, Frankfurt am Main, Germany

**Keywords:** conspiracy theories, paranoia, jumping to conclusions, delusions, intuitive thinking, analytical thinking

## Abstract

**Background:**

The belief in conspiracy theories and paranoid ideation are often treated as almost synonymous. However, there is to date no research concerning shared underlying cognitive underpinnings of belief in conspiracy theories and paranoid ideation. One potential underlying factor could be the well-known jumping to conclusion (JTC) bias, the tendency of persons with delusions to perform hasty decisions that are sometimes based on little evidence. Furthermore, a preference for a more intuitive general thinking style, as opposed to an analytical thinking style, could be an additional underlying cognitive factor of both conspiracy theories and paranoia. Thus, the aim of the present study is to investigate in a large sample of non-clinical individuals whether the JTC-bias is more pronounced in individuals who display a stronger belief in conspiracy theories and whether both are related to a more intuitive thinking preference.

**Methods:**

We assessed the data of 519 non-clinical individuals regarding their respective approval of 20 specific conspiracy theories in an online study. Further, we assessed the JTC-bias by using a computerized variant of the beads task (fish task). Thinking preferences were measured with the Rational-Experiential Interview.

**Results:**

Subjects who displayed the JTC-bias presented a more pronounced belief in conspiracy theories. In addition, gathering little information in the fish task before performing a decision (less draws to decision) was related to a stronger endorsement of conspiracy theories and a more intuitive thinking style (and a less analytic thinking style). Finally, a preference for intuitive thinking predicted a stronger belief in conspiracy theories in a multiple regression analysis.

**Conclusions:**

Our results demonstrate the implication of a preference for an intuitive thinking style accompanied by a propensity to faster decision-making (JTC-bias) as possible cognitive underpinnings of beliefs in conspiracy theories. Furthermore, our study is the first to confirm the notion of the JTC-bias as a reflection of the use of an intuitive thinking style.

## Introduction

Conspiracy theories are typically unverified and sensationalistic interpretations of events that are “self-insulating against disconfirmation” and are “based on weak kinds of evidence” (1). Beliefs in conspiracy theories are associated with the rejection of generally accepted norms, assumptions, and behaviors, e.g., regarding vaccination (2), combating climate change (3) or political participation (4). However, many of these theories are widely disseminated and accepted (5). Despite growing research in this area, little is still known about the cognitive underpinnings of the belief in conspiracy theories.

A plethora of studies indicates that the individual agreement with various specific conspiracy theories is highly intercorrelated (6, 7), even if the specific conspiracy theories contradict each other. For example, people who believed that Princess Diana had been murdered were also more likely to agree that she had also faked her own death (8). This tendency to presume conspiracies as the primary cause of important societal events (8, 9) is defined as *conspiracy belief* (CB).

Numerous polls over the last decades found that conspiracy theories are a common phenomenon, with recent studies suggesting that 63% of the American public believed at least one political conspiracy theory and half of Americans believed at least one medical conspiracy theory (5, 6, 10). The wide prevalence and acceptance of conspiracy theories in the general population suggests that CB does not necessarily indicate a mental disorder (11, 12). Nevertheless, parallels to paranoia, defined as “a tendency on the part of an individual or group toward excessive or irrational suspiciousness and distrustfulness of others” (13) are evident. As Jovan Byford (14) puts it: “The link between conspiracy theories and paranoia has become so strong that the two terms are now treated as almost synonymous.”

However, paranoia or persecutory ideation, as it is found in psychotic disorders, usually involves some form of personal and immediate threat, typically targeted on individual or closely related parties (15). In contrast, CB is less self-referential than paranoia, with broader groups of people or in many cases the whole world being the target of the assumed conspiracy (5). Aside from these important differences, both phenomena imply a deep mistrust in external factors and agents (14, 16). In line with this contention, two studies have demonstrated an association of CB and paranoia in non-clinical samples (17, 18).

Paranoia is a common symptom especially of psychotic disorders and a significant feature of persecutory delusions (19). However, it is notable that persecutory ideation is not solely a clinical phenomenon. As psychotic symptoms also commonly occur in the general population, many researchers argued that there is a continuum between everyday suspicions and clinically relevant delusions (20–23). In line with this contention, it is estimated that up to 15% of the general population regularly experience paranoid thoughts (19). Considering the wide prevalence of conspiracy theories and paranoid thoughts as well as the obvious parallels between both phenomena, it also seems plausible that “deficits and stressors that predispose an individual to conspiracy thinking are similar to, if less intense than, those involved in the etiology of paranoid psychosis” (24). However, despite of the two promising studies mentioned above that found preliminary evidence of an association between paranoia and CB (17, 18), to our knowledge there is no research concerning possible shared underlying cognitive mechanisms of both paranoia and CB.

With respect to said “deficits [ … ] involved in the etiology of paranoid psychosis” (24), important factors in the formation and maintenance of delusions are various cognitive biases or thinking errors (25, 26). Possibly the most commonly studied cognitive bias is the *jumping to conclusions* (JTC) bias (27), defined as the tendency of individuals to make quick decisions sometimes based on little evidence (28). There is converging meta-analytical evidence that people with psychosis tend to display a more extreme reasoning style with generally hastier decision making behavior in comparison to non-clinical controls (29–31). Typically, the JTC-bias is measured with the *beads task* (27). This task involves two jars containing two types of differently colored beads in an opposite ratio (e.g., 85% orange; 15% blue and vice versa). After the jars are presented to the subjects, they are hidden from view and subjects are consecutively presented beads from only one jar. After each round, the subjects are asked if they are able to decide from which jar the beads are drawn. A decision after one or two beads is considered as JTC-bias, while the total number of beads subjects require before deciding is called *draws to decision* (DTD). Given the considerable parallels between CB and paranoia mentioned above, it thus would be interesting to further assess a possible implication of the JTC-bias in CB.

In general, the JTC-bias seems to reflect a broader tendency of individuals to rely on a faster, heuristic thinking style in contrast to a slower, more analytical thinking style (32). These two thinking styles are postulated in several so-called dual process models of human reasoning and typically differ in several key characteristics (33–35). The faster and relatively effortless route of reasoning is typically regarded as independent of cognitive abilities, as well as working memory and relies on heuristics and intuition. The slower and effortful, analytical route relies heavily on the cognitive ability and is dependent on the working memory of the individual. However, although the claim that the JTC-bias might reflect the use of the intuitive thinking style has an intuitive appeal, to our knowledge an association of both constructs has yet to be empirically established.

Regarding the belief in conspiracy theories, as mentioned before, there is a broader tendency to presume conspiracies as the primary cause of important societal events (8, 9). This broader tendency (defined as CB), could in turn reflect a preference for one or the other thinking style described above. As “widespread irrational beliefs often have strong intuitive appeal” (36) and conspiracy theories tend to trigger a strong affective response (37), CB could reflect an individual’s preference for the use of the intuitive thinking style. On the other hand, “[conspiracy] theorists do not see themselves as raconteurs of alluring stories, but as investigators and researchers,” (14) which would consequently imply a preference for a more analytical thinking style. These contradicting considerations echo in current research regarding this topic, as preliminary findings suggest that CB seems to be associated with an increased use of an intuitive thinking style, while the evidence for the relationship with the use of the analytic thinking style remains equivocal (37, 38).

Taken together these findings provide preliminary evidence indicating that the preference for an intuitive, heuristic style of thinking is related to CB. However, in order to derive clear-cut evidence about the role of thinking styles in the formation and maintenance of CB, it is important to further scrutinize this association.

Overall, we consider it most relevant to further investigate the topic of CB regarding possible cognitive underpinnings. While preliminary evidence points at a preference for an intuitive thinking style as the foundation of CB, the role of specific mechanisms of the experiential system (e.g., the JTC-bias) has not been examined before. Consequently, as the JTC-bias is commonly found in subjects suffering from delusions and even in delusion-prone individuals (29–31), we predict first, in line with the notion of shared underlying cognitive mechanisms between paranoia and CB, that persons who display a more pronounced JTC-bias hold a stronger CB (hypothesis 1a) and that less *draws to decision* in a computerized variant of the beads task (fish-task) are negatively correlated with CB (hypothesis 1b).

Regarding the role of the two thinking styles, we first consider it important to validate the – intuitively appealing – idea, that the JTC-bias is a reflection of the use of the experiential system (32). Thus, we hypothesized that subjects who display the JTC-bias show a stronger preference for intuitive thinking as well as an aversion to analytic thinking in comparison to subjects who show a more cautious information gathering style (hypothesis 2a) and that a preference for intuitive thinking (in contrast to analytic thinking) is associated with less *draws to decision* in a computerized variant of the beads task (hypothesis 2b).

Third, our aim was to further elucidate the interplay of the different thinking styles and CB. Most conspiracy theories are intuitively appealing and therefore individuals who exhibit a stronger preference for an intuitive thinking style could, consequently, be more inclined to belief in said theories. As mentioned before, evidence regarding an association of analytical thinking and CB is equivocal, probably because conspiracy theorists see themselves “as investigators and researchers.” (14). However, considering the promising results regarding the role of the use of intuitive thinking, we hypothesized that only the preference for intuitive thinking predicts a stronger CB (hypothesis 3).

## Materials and Methods

### Recruitment and Procedure

Recruitment was accomplished via social media and a survey-sharing platform (surveycircle.com), which allows to disseminate one`s own study in return for participation in other online-studies. An additional incentive was a voluntary lottery with 15 Amazon-vouchers (20 € each). The study was described to potential participants as an investigation of the association between political attitudes and decision behavior (associations between political attitudes and CB will be reported elsewhere).

All participants were first informed about the assessment and gave informed consent, then they saw a 4-min video^1^ explaining the *fish task* described below, as misunderstanding is a common phenomenon regarding the measurement of the JTC-bias (39). Subsequently, participants were directed to the measures described below. Finally, participants answered questions on sociodemographic data. They were then asked whether they had answered all questions conscientiously and truthfully and whether they considered their data as valid. Finally, participants received a summary of their results after the study was completed. More specifically, they received a short overview about the scientific background of the study. Applicable ethical standards of the German Psychological Society (DGPs) were followed, no experimental manipulation took place and anonymity was assured. The requirement of formal ethical review/approval was waived by the Ethics Committee of the University of Marburg (Faculty of Psychology), as no experimental manipulation took place, participants received information on the study, provided written informed consent, and anonymity was assured.

### Subjects

Subjects were included if they had access to social media and their age was above 16. From the originally 519 participants, 30 were excluded either because they declared their data to be invalid (*n* = 7) or because they were considerably faster than the other participants (*z* < −1.96; n = 23). We also excluded one participant who declared to suffer from schizophrenia, as the tendency to jump to conclusions is influenced by the state of remission of the disorder (28, 40, 41).

### Measures

#### Conspiracy Belief

The tendency to endorse conspiracy theories, defined as conspiracy belief (CB), was measured using the classical approach of asking participants to rate several specific conspiracy theories regarding their respective approval (42). The mean approval rate of said theories is then interpreted as a measure for the superordinate general CB. The different conspiracy theories derive from a previous study that examined the popularity and approval of several conspiracy theories in German-speaking countries (43). Thirty conspiracy theories with the most pronounced approval rating were used in a pilot study (44) and from these 30 conspiracy theories, 20 theories with the highest discriminability were selected for the updated assessment of CB The 20 different conspiracy theories are depicted in **Appendix A**. Participants had to read verbal descriptions of the 20 conspiracy theories and were then asked to rate their respective approval of said theories on a five-point Likert scale ranging from 1 (I do not agree at all) to 5 (I fully agree) or to choose “Not able to judge/theory not known” as an answer if they did not want or were able to decide about a specific conspiracy theory. The mean approval rate of all conspiracy theories was used as measure of CB (range, 1–5). The mean approval rate of the total sample was 2.63 (*SD* = .76) and the final questionnaire had an excellent reliability with Cronbach’s α = .94. An overview of all the theories used in the present study and their intercorrelations can be found in **Appendixes A1**, **B1**.

#### Analytic and Intuitive Reasoning

Analytic and intuitive reasoning was measured with the German version of the *Rational-Experimental Inventory* (45). The original version of Epstein, Pacini, Denes-Raj, and Heier (46) is theoretically based on the *Cognitive-Experiential Self-Theory of Personality* (33). The German version of the Rational-Experiential Inventory consists of 29 items measuring the individual’s preferences regarding different types of information processing, subsumed to two subscales (the *Need for Cognition* scale and the *Faith in Intuition* scale). The *Need for Cognition* scale consists of 14 items measuring the preference for an analytical thinking style (derived from the 45-item *Need for Cognition* scale, originally developed by Cacioppo & Petty (47). The *Faith in Intuition* scale consists of 15 items measuring the preference for an intuitive thinking style (range, 15–105). The questions are answered on a seven-point Likert scale ranging from 1 (completely wrong) to 7 (completely true). The two subscales were confirmed factor-analytically, both in the original as well as the German version of the questionnaire (45, 46) The subscales of the German version feature a good reliability with Cronbach’s α = .82 (*Need for Cognition* scale) and α = .86 (*Faith in Intuition* scale).

#### Jumping to Conclusions (JTC)

The JTC-bias was measured with a modified version of the traditional *beads task* (27). The *fish task* uses a more easily understandable scenario of a fisherman who fishes differently colored fish from one of two ponds (48). Participants are first informed that two fish species (orange and blue fish) are located in two ponds (A and B) in reversed ratio, 60 orange and 40 blue fish in pond A and 40 orange and 60 blue fish in pond B. The subjects are furthermore made aware of being presented with fish from only one pond without knowing which pond the fish come from (prior probability of 50 percent). After each fish that is presented, the subjects are asked if they are ready to decide from which pond the fish is drawn. A decision after one or two fish is considered as a JTC-bias. The sequence of fish presented is orange-orange-orange-blue-orange-orange-orange-orange-blue-orange. In the present paradigm, the posterior probability for choosing pond A after the presentation of one orange fish is 60% and for choosing pond A after the presentation of two orange fish is 69%. As an additional JTC-measure, the number of *draws to decision* (DTD) (range, 1–10) is recorded: the number of fishes the subjects views until they decide that the fish stem from one pond.

#### Demographic Form

Participants provided their demographic details, consisting of gender (*man, woman*, and *diverse*), age, mental disorders in the past/present, highest educational qualification, and political affiliation.

### Statistical Analyses

Following the *central limit theorem* (49), independent variables in samples of *n* > 30 can be viewed as sufficiently normally distributed. Thus, as all groups consisted of more than 30 participants, we assumed normal distribution of the variables.

The assumed group difference regarding CB between participants who showed the JTC-bias (indicated by a decision after one or two fishes fished in the fish task) and those who did not (hypothesis 1a) was analyzed by performing an independent-sample *t*-test, if the assumption of homoscedasticity was met, verified by the Levene’s test. In case of heteroscedasticity, degrees of freedom were adjusted accordingly. Possible group differences regarding the two thinking styles (indicated by the *Faith in Intuition* score and the *Need for Cognition* score) between participants who showed the JTC-bias (indicated by a decision after one or two fishes fished in the fish task) and those who did not (hypothesis 2a) were also analyzed by performing two independent-sample *t-*tests, as both thinking styles are theoretically independent. Furthermore, we examined the association between *draws to decision* in the fish task and CB (hypothesis 1b) as well as both thinking styles (indicated by the *Faith in Intuition* score and the *Need for Cognition* score, hypothesis 2b) by using Pearson’s two-tailed correlations.

Finally, a multiple regression analysis was performed using the SPSS-Enter method in order to test if a preference for intuitive thinking predicts CB (hypothesis 3). We included the preference of both an intuitive and an analytical thinking style (indicated by the *Faith in Intuition* score and *Need for Cognition* score respectively) as predictors and CB (mean score) as criterion. The required assumptions (independence of errors and absence of multicollinearity) were tested by calculating the *Durbin-Watson statistic* as well as the *variance inflation factor*.

We repeated all analyses controlling for age and gender by performing an ANCOVA for hypotheses 1a and 2a and a partial correlation analysis for hypotheses 1b and 2b. The multiple regression (hypothesis 3) was conducted by first calculating a regression model only with age and gender as predictor variables and CB (mean score) as criterion, we then included both thinking styles as predictors. Since the inclusion of the relatively small group of 5 “diverse” individuals led to a violation of some statistical assumptions, we performed the additional analyses for age and sex twice, once with and once without the five “diverse” individuals. Gender was dummy-coded in the analysis that included the five individuals. The results of the analyses can be taken from the **Supplement S1**.

## Results

### Sample Characteristics

The final sample consisted of 488 non-clinical individuals of whom 295 identified as women and 198 identified as men and 5 identified as “diverse”. An analysis of variance (ANOVA) yielded significant differences in both analytic thinking (*F*(2,485) = 8.44, *p* <.001) and intuitive thinking (*F*(2,485) = 5.28, *p* = .005) between genders. Post hoc Tukey tests showed men and women differed significantly regarding their thinking preferences, with men (*M* = 73.52, *SD* = 14.15) showing a stronger preference for analytic thinking than women (*M* = 68.73, *SD* = 12.19), *p* <.001. On the other hand, women (*M* = 62.92, *SD* = 11.33) showed a stronger preference for intuitive thinking than men (*M* = 59.33, *SD* = 13.12), *p* = .004. Age ranged between 17 and 70 (*M* = 28.11; *SD* = 7.79) and was significantly correlated (all *p* <.05) with CB (*r* = .11), intuitive thinking (*r* = −.10) and analytic thinking (*r* = .12).

### JTC-Bias and Conspiracy Belief

#### Differences in CB Between Participants With and Without a JTC-Bias (Hypothesis 1a)

Mean scores of agreements with the 20 specific conspiracy theories and their respective intercorrelations are depicted in **Appendix B1**. The level of agreement with the 20 specific conspiracy theories was quite large and intercorrelations between CB were mostly of medium effect size. Ranges of agreement to the individual theories varied considerably, range of the mean score lay between 1 and 5.

As depicted in **Table 1**, we found statistically significant differences between participants who did and did not display the JTC-bias with regard to CB. As *Levene*’*s Test* indicated homogenous variances (*p* = .39), the group comparison was performed as a *t*-test. In comparison to persons who did not jump to conclusions (*M* = 2.58, *SD* = .74), participants who jumped to conclusions (*M* = 2.99, *SD* = .81) showed a significantly higher CB score (*t*(482) = 4.20, *p* <.001, Cohen’s *d* = .53). Controlling for age and gender did not affect the significance of the results (see **S2** and **S8**).

**Table 1:** Comparison of Participants Regarding Cognitive Measures (CB, Thinking Styles) and JTC Measures.

	Total sample (*N* = 488)	JTC yes (*n* = 69)	JTC no (*n* = 419)	Statistics
	*M (SD)*	*M (SD)*	*M (SD)*	
Conspiracy belief	2.66 (*.73*)	2.99 (*.81*)	2.58 (*.74*)	*t*(482) = 4.20, *p* <.001
*Cognitive measures*				
Faith in Intuition Scale	62.00 (*11.75*)	66.41 (*12.48*)	60.67 (*11.97*)	*t*(486) = 3.67, *p* <.001
Need for Cognition Scale	69.83 (*12.70*)	64.78 (*16.27*)	71.59 (*12.43*)	*t*(81.58)* = 3.32, *p* <.001
*JTC measures*				
Draws to decision	4.65 (*2.32*)	1.45 (*.50*)	5.24 (*2.03*)	*t*(379.63)* = 29.32, *p* <.001

JTC, jumping to conclusions.

*as Levene’s Test indicated inequal variances, degrees of freedom were adjusted accordingly.

#### Analysis of the Association Between DTD and CB (Hypothesis 1b)

Results of Pearson’s correlation coefficients suggested a significant correlation between less draws to decision in the fish task and a more pronounced CB, *r*(384) = −.16, *p* = .002, 95% CI [−.26 to −.05]. **Table 2** depicts intercorrelations between all other variables. Controlling for age and gender did not affect the significance of the results (see **S5** and **S11**).

**Table 2:** Associations between Conspiracy Beliefs, Thinking Styles, and the JTC-bias.

		*M*	*SD*	2	3	4
1	Conspiracy belief	2.64	0.77	**0.363** ^***^	−**0.190** ^***^	−**0.160** ^**^
2	Faith in Intuition Score	61.44	12.17		−**0.359** ^***^	−**0.200** ^***^
3	Need for Cognition Score	70.70	13.13			**0.146** ^*^
4	JTC draws to decision	4.56	2.35			

JTC, jumping to conclusions.

Significant correlations are written in bold.

^*^ p = 0.004.

^**^ p = 0.002.

^***^ p < 0.001.

### JTC-Bias and the Information Processing Systems

#### Differences in the Use of Intuitive and Analytical Thinking Between Participants With and Without a JTC-Bias (Hypothesis 2a)

As Levene’s tests indicated homogenous variances for the *Faith in Intuition* score (*p* = .72), the group comparison was performed as a *t*-test for independent groups. Participants who presented a JTC-bias (defined as a decision after one or two fishes in the fish task, *n* = 69) showed a significantly higher *Faith in Intuition* score (*M* = 66.41, *SD* = 12.48) than those who did not jump to conclusions (*n* = 419, *M* = 60.67, *SD* = 11.97), with *t*(486) = 3.67, *p* <.001, Cohen’s *d* = .47). In case of the *Need for Cognition* score, Levene’s test indicated heterogenous variances (*p* <.001). Consequently, the degrees of freedom were adjusted accordingly. Participants who showed a more pronounced tendency to jump to conclusions showed a significantly lower *Need for Cognition* score (*M* = 64.78, *SD* = 16.27) than those who did not jump to conclusions (*M* = 71.59, *SD* = 12.43), *t*(81.58) = 3.32, *p* = .001, Cohen’s *d* = .47). Controlling for age and gender did not affect the significance of the results (see **S3**, **S4**, **S9** and **S10**).

#### Analysis of the Association Between DTD and Intuitive and Analytical Thinking (Hypothesis 2b)

Results of the correlation analysis revealed a statistically significant correlation between a lower number of *draws to decision* (DTD) in the fish task and a more pronounced *Faith in Intuition* score, (*r*(384) = −.20, *p* <.001, 95% CI [−.30, −.10]). In addition, results revealed a significant association between DTD and a more pronounced *Need for Cognition* score (*r*(384) = .15, *p* = .004, 95% CI [.05,.24]). Controlling for age and gender did not affect the significance of the results (see **S5** and **S11**).

#### Intuitive Thinking as a Predictor of Conspiracy Belief (Hypothesis 3)

A standard multiple linear regression analysis using the enter method indicated that CB (criterion variable) was significantly predicted by the *Faith in Intuition* score (β = 0.34, *t*(483) = 7.45, *p* <.001), whereas the *Need for Cognition* score was not a statistically significant predictor of CB. (β = −.07, *t*(483) = −1.51, *p* =.13). The model explained 14% of the variance of the CB-variable, with *F*(2, 481) = 37.79; *p* <.001; *R^2^* = .14. When controlling for age and gender, we found that age was also a significant predictor of CB (β = 0.16, *t*(478) = 3.71, *p* <.001) (see S6 and S12).

## Discussion

The present study yielded some interesting results. Participants who displayed the *jumping to conclusions* (JTC) bias were more likely to endorse conspiracy theories than subjects who did not jump to conclusions, thus presented a stronger generalized *conspiracy belief* (CB). In addition, subjects who required more *draws to decision* (DTD) in the fish-task presented a less pronounced CB. Moreover, less DTD correlated significantly with a preference for a more intuitive thinking style (indicated by the *Faith in Intuition* score) and, in line with this, subjects who displayed the JTC-bias showed a significantly stronger preference for an intuitive thinking style than subjects who did not jump to conclusions. The opposite pattern of results was found in case of the preference for an analytical thinking style (indicated by the *Need for Cognition* score). Finally, the preference for intuitive thinking (in contrast to a preference for analytical thinking) predicted CB. Moreover, age was also a significant predictor of CB.

First and foremost, we were especially interested in the question of whether the conceptual similarities between CB and paranoia (16) are predicated on the same psychological mechanisms. While the correlation between DTD in the fish task and CB was of small effect size (*r* = −.16), we found a medium effect size (Cohen’s *d* = .53) regarding the group differences between subjects who jumped to conclusions and those who did not. This implies that, while there is a rather small general association between decision-making behavior and CB, especially those subjects with an extreme reasoning style (a decision after two or less fish in the fish task) differ considerably from the rest of the sample regarding their overall CB. Consequently, our findings provide preliminary evidence for an implication of cognitive mechanisms (in this case, the JTC-bias) in CB that are typically linked to paranoia or delusional ideation (19).

Moreover, to our knowledge, our study is the first to present empirical evidence for an association of the JTC-bias and a preference for the use of a more intuitive thinking, as indicated by the correlation of DTD and the *Faith in Intuition* score as well as the significantly higher *Faith in Intuition* score in subjects who displayed the JTC-bias in the fish task. This is in line with the theoretical integration of Ward and Garety (32) who proposed that the JTC-bias might be an aspect of a broader tendency of an over-reliance on faster, heuristic reasoning processes. Consequently, the opposite pattern of results was found in case of a preference for analytical thinking. However, as our study is to our knowledge the first to show an association between the preference for an intuitive thinking style and the JTC-bias, our findings should be regarded as preliminary and require careful pre-registered replication in different samples and with different measures of JTC.

Furthermore, we set out to provide further evidence for the relevance and importance of a preference for a more intuitive thinking style regarding the formation of CB. In fact, results of the regression analysis indicate that a preference for intuitive thinking predicts the degree of CB, with a moderate effect size of *R*^2^ = .14 (50). Simply said, a greater trust in one’s own intuition (in conjunction with a propensity to jump to conclusions) leads to a faster acceptance of the “simple yet satisfying narratives” (51) which are found in many conspiracy theories. In contrast, it may require considerably more cognitive effort (which would, on the other hand, require a preference for analytic thinking) or it might be virtually impossible to retrace the impact of a complex network of individual agents, each pursuing their own agenda (52). As a consequence, people with a preference for intuitive thinking might tend to heuristically conflate these agents and thus assume that only a small group of individuals “is pulling the strings” (53) behind actions that could be perceived as coordinated. Additionally, conspiracy theories tend to trigger a stronger affective response, which in turn makes them more likely to appeal to persons who prefer an intuitive thinking style (37). This finding is in line with prior results indicating a close association of both constructs (37, 38).

Regarding the association of CB and analytic thinking, our results are in line with another study that did not find a significant association of both constructs (38). Since many authors count CB to other “stigmatized knowledge” (54) like esoteric or superstitious ideas, it may seem counter-intuitive that, on the other side, a preference for analytical thinking does not predict a less pronounced CB (as analytical thinking correlates negatively for example with religious or paranormal beliefs (55)). Surprisingly, interventions aiming at promoting analytic thinking were successful in reducing CB (37). Consequently, these contrasting findings warrant an explanation. As Jovan Byford puts it: “Conspiracy theorists do not see themselves as raconteurs of alluring stories, but as investigators and researchers.” (14). As mentioned above, conspiracy theories are per definition “epistemically self-insulating against disconfirmation” (1) and strong analytic reasoning skills might even be used to generate ideology-consistent interpretations of evidence that are inconsistent with personal beliefs (36). Consequently, a preference for analytic thinking may not detract from or, in some cases, could actually benefit the maintenance of CB (38). Future studies could therefore scrutinize the role of possible moderators (e.g., ideology or political beliefs) of the association of analytic thinking and CB.

Another possible explanation concerns the measurement of both thinking styles: As the *Rational Experiential Inventory* (46) and its German version (45) used in the present study are both self-assessments, they could measure rather the self-perception of the participants than the actual thinking preferences the participants use more often in their daily life. In this regard, subjects that present a pronounced CB might see themselves “as investigators and researchers.” (14) and therefore report a stronger preference for analytical thinking, while actually thinking rather intuitively. Consequently, future research could be improved by incorporating other, more objective measures of intuitive and analytic thinking, such as the *Cognitive Reflection Test* (56) or experimental paradigms that require participants to use either a more intuitive or analytical thinking style. However, despite the lack of clarity regarding the individual’s actual use of the two styles of thinking, there is now in either case growing evidence that a greater faith in one’s own intuition goes hand in hand with a stronger belief in conspiracy theories.

Interestingly, the age of the participants was also a significant predictor of CB. It may be that as people grow older, they may experience more political scandals, which in turn undermines their general trust in established institutions such as politics and the media and thus fuels CB (54). This finding could also indicate that other socio-demographic and socio-economic factors (e.g., formal education or income) could also contribute to the formation of CB, as “such beliefs confirm the person’s sense that the world is beyond their control, while also protecting self-esteem by offering a simple explanation for existential and status-related problems” (14). This question should be examined more closely in future studies.

As to possible clinical implications of our findings, we would like to point out again that believing in conspiracy theories per se is not a mental disorder (11, 12). Real political conspiracies, such as the Iran Contra Affair or the Watergate Scandal (14) recurred throughout history and it would not have been possible to uncover them without a certain degree of mistrust of official institutions and narratives. On the other hand, CB is accompanied by a wide range of negative medical or societal effects such as a reduced willingness to vaccinate (2) or to combat climate change (3) and decreased political participation (4). Consequently, interventions aimed at reducing CB have increasingly come into the focus of conspiracy theory research. Psychological interventions like priming (37) or inoculation (57) showed preliminary, but promising results in reducing CB. If replicated, especially the high propensity to display the JTC-bias in individuals who present a pronounced CB could be the basis for new interventions aiming at reducing CB, as there are already well-established approaches that proved successful in reducing the tendency to jump to conclusions, like the *Metacognitive Training* (58) or *Cognitive Behavior therapy for psychosis* (59).

The present study has some notable features. First, based on a large sample of non-clinical individuals, our study is the first to provide empirical evidence for the notion that the JTC-bias might reflect a preference for intuitive thinking. Second, we were able to shed some light on the possible underpinnings of CB by highlighting that CB is most likely based upon similar cognitive mechanisms as paranoid ideation or delusions, in this case the JTC-bias. Accordingly, CB is predicted by a preference for intuitive thinking.

In interpreting the findings of our study, some limitations should be considered. First, our study was conducted as an online study. As none of assessments was performed face-to-face, subjects were not able to ask for additional information in case of difficulties of understanding. As miscomprehension is a common problem regarding JTC- tasks such as the beads task or the fish task (39), we tried to address this problem by creating a 4-min-long video with the purpose of explaining the fish task in an easily understandable manner. However, watching the video was not obligatory and we did not check for possible misunderstandings of the task. Additionally, the online survey included a lottery. We cannot rule out the possibility that some subjects only took part for the purpose of participation in the lottery and did not answer carefully. We tried to minimize this risk by asking the subjects after the experimental procedure if they considered their data to be valid and whether we could use their data. Referring to this, we emphasized that the answer would not affect the participation in the lottery. While only seven participants advised us against using their data, possibly some subjects may not have answered this question truthfully. Finally, another limitation concerns the exclusion of certain individuals. One participant was excluded because he declared to suffer from schizophrenia. Although it would certainly be exciting to investigate the extent to which schizophrenia, especially persecutory delusions, and CB are related, the focus of this study was supposed to be particularly on CB in non-delusional individuals.

Another limitation of our study should be addressed by future research: although experimental manipulation is not imperative to claim causality (in this case, assuming that a preference for intuitive thinking predicts CB (60)), future research could involve experimental paradigms in order to gain stronger evidence of the assumed causality of the constructs. Additionally, despite the advantages of online research (e.g., economy and a stronger feeling of anonymity (61)), our results should be verified in a traditional face-to-face setting. Furthermore, future studies are well-advised to pre-register their hypotheses and analyses, as studies that are not preregistered tend to overestimate effects (62). Finally, building on our promising results, future studies concerned with CB could scrutinize the role of other parameters involved in the formation and maintenance of paranoid ideation (e.g., belief flexibility or affective states, e.g., loneliness (26)).

In conclusion, we were able to shed some light on the cognitive underpinnings of conspiracy beliefs. More specifically, the results of the present study indicate that a preference for an intuitive thinking style, accompanied by a propensity to jump to conclusions, might be an important factor in the formation of conspiracy beliefs. In a nutshell, although the belief in conspiracy theories reflects by no means a mental disorder, it is possibly associated with the same cognitive processes as paranoid ideation or delusions.

## Data Availability Statement

The dataset is available on this paper's project page on the OSF: https://osf.io/er374/.

## Ethics Statement

The requirement of formal ethical review/approval of the studies involving human participants was waived by the Ethics Committee of the University of Marburg (Faculty of Psychology), as no experimental manipulation took place, participants received information on the study, provided written informed consent, and anonymity was assured.

## Author Contributions

NP and SM contributed to conception and design of the study. NP organized the data analysis and performed the statistical analysis. NP wrote the first draft of the manuscript. DS and SM wrote sections of the manuscript. All authors contributed to the article and approved the submitted version.

## Conflict of Interest

The authors declare that the research was conducted in the absence of any commercial or financial relationships that could be construed as a potential conflict of interest.

## Supplementary Material

The Supplementary Material for this article can be found online at: https://www.frontiersin.org/articles/10.3389/fpsyt.2020.568942/full#supplementary-material



## Footnotes

1
https://youtu.be/YuJuCqdTBgs

## Overview about the specific conspiracy theories used in the present study

English translation of the conspiracy theories used in the present study

I think…

1. J. F. Kennedy was not shot by Lee Harvey Oswald (alone).
2. Scientology has great influence in the federal republic of Germany; various large companies belong to Scientology.
3. in the former Union of Soviet Socialists Republic (USSR) there were several serious covered-up nuclear power accidents.
4. the true story behind the attacks of 11 September 2001 does not correspond to the version disseminated by the Bush government.
5. influential Jewish families control large parts of world affairs.
6. Lady Di (Diana of Wales) was murdered.
7. the USA invaded Iraq in 2003 in order to gain access to oil.
8. for some time now, various governments have had contact with aliens.
9. there is a secret society of “Illuminati” whose symbols are the All Seeing Eye, the pyramid and the number “23”.
10. airplane condensation trails are in reality secret experiments, so-called “chemtrails”, which damage the environment.
11. Jesus and Mary Magdalene fathered children, which is being covered up by the Church.
12. the World Trade Center collapsed mainly because it was blown up from inside.
13. there are various religious groups that perform human sacrifices.
14. the automotive industry is only abandoning the use of stainless steel in exhaust systems because their regular replacement would jeopardize sales.
15. there are religious sects that have complete control over the psyche of their members.
16. behind various events in world history are actually the Freemasons.
17. the pharmaceutical industry blocks the distribution of certain useful drugs.
18. the Nazis developed functioning flying discs in UFO-optic during World War II.
19. in the US there were several serious nuclear accidents that have been covered-up.
20. a small group of people directs the fate of the Earth.

*Original German Items*

Ich denke, …

1. J. F. Kennedy wurde nicht von Lee Harvey Oswald (allein) erschossen.
2. Scientology besitzt großen Einfluss in der BRD; verschiedene Großunternehmen gehören zu Scientology.
3. in der ehemaligen UDSSR gab es mehrere schwere vertuschte Atomkraftunfälle.
4. die wahre Geschichte hinter den Anschlägen vom 11. September 2001 entspricht nicht der von der Bush - Regierung verbreiteten Version.
5. einflussreiche jüdische Familien kontrollieren große Bereiche des Weltgeschehens.
6. Lady Di (Diana von Wales) wurde ermordet.
7. die USA sind wegen des Öls im Jahr 2003 in den Irak einmarschiert.
8. seit längerer Zeit haben verschiedene Regierungen Kontakt zu Außerirdischen.
9. es gibt einen Geheimbund der “Illuminaten”, deren Symbole das Allsehende Auge, die Pyramide und die Zahl “23” sind.
10. Flugzeug-Kondensstreifen sind ab und an in Wirklichkeit Geheimversuche, sogenannte “Chemtrails”, die die Umwelt schädigen.
11. Jesus hat mit Maria Magdalena Kinder gezeugt, was von der Kirche vertuscht wird.
12. das World Trade Center stürzte vor allem ein, weil es von innen gesprengt wurde.
13. es gibt verschiedene religiöse Gruppen, die Menschenopfer durchführen.
14. die Automobilindustrie verzichtet auf den Einsatz von rostfreiem Stahl bei Auspuffanlagen nur deshalb, weil das die Umsätze mit deren regelmäßigem Austausch gefährden würde.
15. es gibt religiöse Sekten, die die vollständige Kontrolle über die Psyche ihrer Mitglieder haben.
16. hinter verschiedenen Geschehnissen der Weltgeschichte stehen in Wirklichkeit die Freimaurer.
17. die Pharmaindustrie blockiert die Verbreitung gewisser sinnvoller Medikamente.
18. die Nazis haben im Zweiten Weltkrieg funktionierende Flugscheiben in UFO-Optik entwickelt.
19. In der USA gab es mehrere schwere vertuschte Atomkraftunfälle.
20. eine kleine Gruppe von Personen lenkt die Geschicke der Erde.

## Appendix B

**Table B1:** Intercorrelations between the specific conspiracy theories.

	Theory	M	SD	2	3	4	5	6	7	8	9	10	11	12	13	14	15	16	17	18	19	20
1	JFK	2.95	1.22	**.224**	**.190**	**.444**	**.372**	**.328**	**.240**	**.266**	**.293**	**.299**	**.250**	**.396**	**.129**	**.205**	.066	**.265**	**.229**	**.343**	**.204**	**.234**
2	Scientology	2.25	1.18		**.438**	**.304**	**.403**	**.366**	**.167**	**.308**	**.325**	**.365**	**.138**	**.330**	**.234**	**.252**	**.291**	**.430**	**.306**	**.440**	**.472**	**.286**
3	Nuclear Accidents UDSSR	2.89	1.19			**.282**	**.288**	**.262**	**.132**	**.247**	**.265**	**.226**	**.168**	**.250**	**.268**	**.284**	**.251**	**.321**	**.318**	**.332**	**.601**	**.239**
4	9/11	2.83	1.34				**.472**	**.451**	**.373**	**.371**	**.375**	**.383**	**.155**	**.681**	**.305**	**.348**	**.216**	**.395**	**.391**	**.405**	**.406**	**.406**
5	Jewish Conspiracy	2.01	1.24					**.450**	**.339**	**.409**	**.529**	**.552**	**.160**	**.507**	**.347**	**.302**	**.219**	**.651**	**.363**	**.535**	**.387**	**.575**
6	Lady Die	2.62	1.32						**.279**	**.344**	**.312**	**.338**	**.127**	**.497**	**.352**	**.331**	**.214**	**.407**	**.263**	**.405**	**.417**	**.354**
7	Iraq War	3.83	0.99							**.150**	**.234**	**.189**	**.217**	**.360**	**.174**	**.358**	**.197**	**.345**	**.368**	**.197**	**.233**	**.379**
8	Aliens	1.46	0.91								**.515**	**.554**	**.183**	**.452**	**.286**	**.177**	.080	**.494**	**.296**	**.528**	**.338**	**.360**
9	Illuminati	2.29	1.33									**.503**	**.262**	**.492**	**.404**	**.318**	.078	**.640**	**.360**	**.449**	**.256**	**.450**
10	Chemtrails	1.46	0.97										**.141**	**.517**	**.256**	**.228**	.059	**.548**	**.311**	**.559**	**.332**	**.482**
11	Jesus’ Offspring	2.62	1.35											**.236**	**.245**	**.245**	.042	**.193**	**.230**	**.175**	**.151**	**.197**
12	WTC Detonation	2.16	1.35												**.366**	**.363**	**.139**	**.482**	**.341**	**.416**	**.351**	**.478**
13	Human Sacrifices	3.45	1.16													**.349**	**.297**	**.413**	**.331**	**.285**	**.374**	**.317**
14	Automotive Industry	3.23	1.16														**.245**	**.311**	**.514**	**.276**	**.414**	**.373**
15	Mind Control	3.95	1.14															**.256**	**.238**	**.188**	**.252**	**.199**
16	Freemasons	2.07	1.14																**.425**	**.493**	**.382**	**.498**
17	Pharma Industry	3.58	1.26																	**.357**	**.442**	**.436**
18	Nazi UFOs	1.82	1.18																		**.436**	**.430**
19	Nuclear Accidents USA	2.27	1.11																			**.406**
20	World Conspiracy	2.40	1.37																			

Significant correlations are written in bold.
