# Corrigendum: Thinking Preferences and Conspiracy Belief: Intuitive Thinking and the Jumping to Conclusions-Bias as a Basis for the Belief in Conspiracy Theories

**DOI:** 10.3389/fpsyt.2021.664972

**Published:** 2021-03-08

**Authors:** Nico Pytlik, Daniel Soll, Stephanie Mehl

**Affiliations:** ^1^Department of Psychiatry and Psychotherapy & Center for Mind, Brain and Behavior (CMBB), Philipps-University, Marburg, Germany; ^2^Department of Health and Social Work, Frankfurt University of Applied Sciences, Frankfurt am Main, Germany

**Keywords:** conspiracy theories, paranoia, jumping to conclusions, delusions, intuitive thinking, analytical thinking

In the original article, there was a mistake in Table 1 and Table 2 as published. In the first row of Table 1 (Conspiracy belief), results were switched for the **JTC yes** column and **JTC no** column. The correct data for Conspiracy belief under the **JTC yes** column is “*M* = 2.99 (*SD* =.81)” and under the **JTC no** column the correct data is “*M* = 2.58 (*SD* =.74)”. The corrected Table 1 appears below.

**Table 1:** Comparison of Participants Regarding Cognitive Measures (CB, Thinking Styles) and JTC Measures.

	**Total sample (*N* = 488)**	**JTC yes (*n* = 69)**	**JTC no (*n* = 419)**	
	***M* (*SD*)**	***M* (*SD*)**	***M* (*SD*)**	**Statistics**
Conspiracy Belief	2.66 (*.73*)	2.99 (*.81*)	2.58 (*.74*)	*t*(482) = 4.20, *p* < .001
*Cognitive measures*				
Faith in Intuition Scale	62.00 (*11.75*)	66.41 (*12.48*)	60.67 (*11.97*)	*t*(486) = 3.67, *p* < .001
Need for Cognition Scale	69.83 (*12.70*)	64.78 (*16.27*)	71.59 (*12.43*)	*t*(81.58)^*^= 3.32, *p* < .001
*JTC measures*				
Draws to decision	4.65 (*2.32*)	1.45 (*.50*)	5.24 (*2.03*)	*t*(379.63)^*^= 29.32, *p* < .001

*JTC, jumping to conclusions*.

* *As Levene's Test indicated inequal variances, degrees of freedom were adjusted accordingly*.

**Table 2:** Associations between Conspiracy Beliefs, Thinking Styles and the JTC-bias.

		***M***	***SD***	**2**	**3**	**4**
1	Conspiracy Belief	2.64	0.77	0.363^***^	-0.190^***^	-0.160^**^
2	Faith in Intuition Score	61.44	12.17		-0.359^***^	-0.200^***^
3	Need for Cognition Score	70.70	13.13			0.146^*^
4	JTC Draws to Decision	4.56	2.35			

*JTC, Jumping to conclusions*.

*Significant correlations are written in bold*.

* *p = 0.004*.

** *p = 0.002*.

*** *p <0.001*.

Additionally, in the third row of Table 2 (Need for Cognition Score), the algebraic sign of the data of column 4, **JTC draws to decision**, is wrong. The correct data for the Need for Cognition Score under the **JTC draws to decision** column is “*r* = 0.146”. The corrected Table 2 appears below.

The authors apologize for these errors and state that they do not change the scientific conclusions of the article in any way. The original article has been updated.

