# Corrigendum: Thinking preferences and conspiracy belief: intuitive thinking and the jumping to conclusions-bias as a basis for the belief in conspiracy theories

**DOI:** 10.3389/fpsyt.2024.1366548

**Published:** 2024-02-29

**Authors:** Nico Pytlik, Daniel Soll, Stephanie Mehl

**Affiliations:** ^1^ Department of Psychiatry and Psychotherapy & Center for Mind, Brain and Behavior (MCMBB), Philipps-University, Marburg, Germany; ^2^ Department of Health and Social Work, Frankfurt University of Applied Sciences, Frankfurt am Main, Germany

**Keywords:** conspiracy theories, paranoia, jumping to conclusions, delusions, intuitive thinking, analytical thinking

In the published article, there was an error. In **Materials And Methods**, *Recruitment and procedures*, paragraph 2, the following text was included: “The local ethics committee approved of the study. Participants provided informed consent and were debriefed after the completion of the study.” The corrected statement appears below:

“The requirement of formal ethical review/approval was waived by the Ethics Committee of the University of Marburg (Faculty of Psychology), as no experimental manipulation took place, participants received information on the study, provided written informed consent, and anonymity was assured.”

Consequently, there was also an error in the **Ethics** statement. The correct statement appears below:

“The requirement of formal ethical review/approval of the studies involving human participants was waived by the Ethics Committee of the University of Marburg (Faculty of Psychology), as no experimental manipulation took place, participants received information on the study, provided written informed consent, and anonymity was assured.”

The authors apologize for these errors and state that this does not change the scientific conclusions of the article in any way. The original article has been updated.

