# Supplementary material for: Thinking Preferences and Conspiracy Belief: Intuitive Thinking and the Jumping to Conclusions-Bias as a Basis for the Belief in Conspiracy Theories
Source: Front Psychiatry. 2020 Sep 18;11:568942. doi: 10.3389/fpsyt.2020.568942 (PMC7530244; doi:10.3389/fpsyt.2020.568942)
Supplement: Supplementary file 1 [file DataSheet_1.pdf]

Supplement

Thinking preferences and conspiracy belief: intuitive thinking and the jumping to conclusions-bias as a basis for the belief in conspiracy theories

This supplement contains supporting information for the above-mentioned manuscript. The results of complementary analyses are reported, the findings are discussed in the main document.

Additional Analyses: Controlling for Age and Gender (men vs. women)

2 **Table S1.** *Means and Standard Deviations of Cognitive Measures (CB, Thinking Styles) and JTC Measures*

2 **Table S2.** *ANCOVA Results for Comparisons of Participants Regarding Conspiracy Belief*

2 **Table S3.** *ANCOVA Results for Comparisons of Participants Regarding their Preference for Intuitive Thinking (FI-score)*

2 **Table S4.** *ANCOVA Results for Comparisons of Participants Regarding their Preference for Analytic Thinking (NC-score)*

3 **Table S5.** *Associations between Conspiracy Belief, Thinking Styles and the JTC-bias controlling for Age and Gender*

3 **Table S6.** *Results of the Multiple Regression Analysis controlling for Age and Gender*

Additional Analyses: Controlling for Age and Gender (total sample)

4 **Table S7.** *Means and Standard Deviations of Cognitive Measures (CB, Thinking Styles) and JTC Measures*

4 **Table S8.** *ANCOVA Results for Comparisons of Participants Regarding Conspiracy Belief*

4 **Table S9.** *ANCOVA Results for Comparisons of Participants Regarding their Preference for Intuitive Thinking (FI-score)*

4 **Table S10.** *ANCOVA Results for Comparisons of Participants Regarding their Preference for Analytic Thinking (NC-score)*

5 **Table S11.** *Associations between Conspiracy Belief, Thinking Styles and the JTC-bias controlling for Age and Gender*

5 **Table S12.** *Results of the Multiple Regression Analysis controlling for Age and Gender*

Results of the additional analyses controlling for age and gender (including only men and women)

Table S1.
Means and Standard Deviations of Cognitive Measures (CB, Thinking Styles) and JTC Measures

	Total sample (N = 479)	JTC yes (n = 67)	JTC no (n = 412)
	M (SD)	M (SD)	M (SD)
Conspiracy Belief	2.63 (*.76*)	2.58 (*.74*)	2.98 (*.81*)
Cognitive measures			
Faith in Intuition Scale	61.45 (*12.21*)	66.26 (*12.51*)	60.66 (*11.99*)
Need for Cognition Scale	70.69 (*13.22*)	65.10 (*16.17*)	71.61 (*12.46*)
JTC measures			
Draws to decision	4.56 (*2.35*)	1.44 (*.50*)	5.24 (*2.04*)

Note: JTC = jumping to conclusions

Table S2.
ANCOVA Results for Comparisons of Participants Regarding Conspiracy Belief

Results	*df*	*SS*	*MS*	*F*	*p*	*partial* η^2
Age	1	3.000	3.000	5.357	.021	.011
Gender	1	1.520	1.520	2.714	.100	.006
JTC	1	7.903	7.903	14.113	<.001	.029
Error	475	265.993	.560			
Total	479	3602.543				

Note: JTC = jumping to conclusions; SS = sum of squares; MS = mean square.

Table S3.
ANCOVA Results for Comparisons of Participants Regarding their Preference for Intuitive Thinking (FI-score)

Results	*df*	*SS*	*MS*	*F*	*p*	*partial* η^2
Age	1	722.870	722.870	5.092	.024	.011
Gender	1	1050.262	1050.262	7.398	.007	.015
JTC	1	1845.452	1845.452	12.999	<.001	.026
Error	479	68003.251	141.969			
Total	483	1895569.000				

Note: JTC = jumping to conclusions; SS = sum of squares; MS = mean square.

Table S4.
ANCOVA Results for Comparisons of Participants Regarding their Preference for Analytic Thinking (NC-score)

Results	*df*	*SS*	*MS*	*F*	*p*	*partial* η^2
Age	1	1103.572	1103.572	6.750	.010	.014
Gender	1	1943.451	1943.451	11.887	.001	.024
JTC	1	2462.353	2462.353	15.061	<.001	.030
Error	479	78314.892	163.497			
Total	483	2497970.000				

Note: JTC = jumping to conclusions; SS = sum of squares; MS = mean square.

Table S5.

Associations between Conspiracy Belief, Thinking Styles and the JTC-bias controlling for Age and Gender

		M	*SD*	2	3	4
1	Conspiracy Belief	2.63	0.76	0.361, $p<.001$	-0.181, $p<.001$	-0.143, $p=.005$
2	Faith in Intuition Score	61.41	12.18		-0.354, $p<.001$	-0.198, $p<.001$
3	Need for Cognition Score	70.77	13.11			0.136, $p=.008$
4	JTC Draws to Decision	4.56	2.36			

Note. JTC = Jumping to conclusions

Table S6.

Results of the Multiple Regression Analysis controlling for Age and Gender

		Unstandardized coefficients		*Standardized coefficients*					
Step	Predictor	*B*	*SE*	*β*	*p*	R^2	R^2 *change*	*F*	*p*
1						.020	.020	4.764	.009
	Age	.012	.004	.120	.009				
	Gender	.137	.071	.088	.055				
2						.158	.138	38.958	<.001
	Age	.015	.004	.159	<.001				
	Gender	.040	.067	.026	.556				
	FI-score	.022	.003	.348	<.001				
	NC-score	-.004	.003	-.073	.112				

Note: *SE* = standard error of B; FI = Faith in Intuition; NC = Need for Cognition

Results of the additional analyses controlling for age and gender (total sample)

Table S7.
Means and Standard Deviations of Cognitive Measures (CB, Thinking Styles) and JTC Measures

	Total sample (*N* = 488)	JTC yes (*n* = 69)	JTC no (*n* = 419)
	M (*SD*)	*M* (*SD*)	*M* (*SD*)
Conspiracy Belief	2.64 (*.77*)	2.58 (*.74*)	2.99 (*.81*)
Cognitive measures			
Faith in Intuition Scale	61.48 (*12.20*)	66.41 (*12.48*)	60.67 (*11.97*)
Need for Cognition Scale	70.63 (*13.24*)	64.78 (*16.27*)	71.59 (*12.43*)
JTC measures			
Draws to decision	4.56 (*2.35*)	1.45 (*.50*)	5.24 (*2.03*)

Note: JTC = jumping to conclusions

Table S8.
ANCOVA Results for Comparisons of Participants Regarding Conspiracy Belief

Results	*df*	*SS*	*MS*	*F*	*p*	*partial* η^2
Age	1	3.141	3.141	5.620	.018	.012
Gender	1	2.120	2.120	3.793	.052	.008
JTC	1	8.339	8.339	14.917	<.001	.030
Error	480	268.315	.559			
Total	484	3653.852				

Note: JTC = jumping to conclusions; SS = sum of squares; MS = mean square.

Table S9.
ANCOVA Results for Comparisons of Participants Regarding their Preference for Intuitive Thinking (FI-score)

Results	*df*	*SS*	*MS*	*F*	*p*	*partial* η^2
Age	1	730.146	730.146	5.162	.024	.011
Gender	1	1089.429	1089.429	7.703	.006	.016
JTC	1	1959.371	1959.371	13.853	<.001	.028
Error	484	68455.520	141.437			
Total	488	1916847.000				

Note: JTC = jumping to conclusions; SS = sum of squares; MS = mean square.

Table S10.
ANCOVA Results for Comparisons of Participants Regarding their Preference for Analytic Thinking (NC-score)

Results	*df*	*SS*	*MS*	*F*	*p*	*partial* η^2
Age	1	1103.112	1103.112	6.760	.010	.014
Gender	1	2141.265	2141.265	13.123	<.001	.026
JTC	1	2720.652	2720.652	16.673	<.001	.033
Error	484	78976.717	163.175			
Total	488	2519604.000				

Note: JTC = jumping to conclusions; SS = sum of squares; MS = mean square.

Table S11.

Associations between Conspiracy Belief, Thinking Styles and the JTC-bias controlling for Age and Gender

		M	*SD*	2	3	4
1	Conspiracy Belief	2.64	0.77	0.370, *p*<.001	-0.192, *p*<.001	-0.143, *p*=.005
2	Faith in Intuition Score	61.44	12.17		-0.333, *p*<.001	-0.198, *p*<.001
3	Need for Cognition Score	70.70	13.13			0.139, *p*=.006
4	JTC Draws to Decision	4.56	2.36			

Note. JTC = Jumping to conclusions

Table S12.

Results of the Multiple Regression Analysis controlling for Age and Gender

		Unstandardized coefficients		*Standardized coefficients*					
Step	Predictor	*B*	*SE*	*β*	*p*	R^2	R^2 *change*	*F*	*p*
1						.024	.024	3.890	.009
	Age	.012	.004	.120	.008				
	Male [1]	-.537	.344	-.345	.119				
	Female [1]	-.400	.343	-.258	.243				
2						.163	.139	39.772	<.001
	Age	.016	.004	.158	<.001				
	Male [1]	-.374	.320	-.240	.243				
	Female [1]	-.336	.318	-.217	.292				
	FI-score	.022	.003	.346	<.001				
	NC-score	-.005	.003	-.078	.087				

Note. *SE* = standard error of B; FI = Faith in Intuition; NC = Need for Cognition, 1 = gender was dummy-coded and subdivided into two genders, persons defining as male would be coded as male = 1 and female = 0, persons defining themselves as female would be coded as male = 0 and female = 1, persons defining themselves as diverse were coded as male = 0, female = 0
